# Sharing Knowledge With Young and Established Students of Immunology by the Neapolitan Gulf at the Ruggero Ceppellini Advanced School

**DOI:** 10.3389/fimmu.2020.00043

**Published:** 2020-01-28

**Authors:** Francesco Colucci

**Affiliations:** ^1^Department of Obstetrics and Gynaecology, National Institute for Health Research Cambridge Biomedical Research Centre, University of Cambridge School of Clinical Medicine, University of Cambridge, Cambridge, United Kingdom; ^2^Centre for Trophoblast Research, University of Cambridge, Cambridge, United Kingdom

**Keywords:** pregnancy, immunity, transplantation, cancer, collaborations

## Introduction

In his *Origin*, Charles Darwin led the foundations to debunk the long-held belief that man and animals derive from separate lineages, landing the final blow in *The Descent of Man*. The discovery in the mid-1980's that fertilized mammal eggs must have male components to generate healthy offspring had similarly dramatic consequences on other religious beliefs, as discussed in “*Genetics: immaculate misconception*” (1). In the Catholic calendar, the 8th of December is dedicated to the Virgin Mary. The occasion was celebrated with loud fireworks cracking during the second night of the 25th course of the EFIS-EJI Ruggero Ceppellini Advanced School of Immunology, held at Castellammare di Stabia, near Naples, 7th−9th December 2014. A faculty of 13 gathered together with 60 attendants from 19 countries to discuss the theme *Maternal Immune System in Pregnancy*. While the conclusions of the course were not quite as dramatic as Darwin's and Surani's, new exciting concepts were discussed that had already emerged at a previous meeting held in Cambridge in 2013 to celebrate the 60th anniversary of Peter Medawar's famous article on the “immunological paradox” of pregnancy (2). I had the honor of directing both events, together with Ashley Moffett, and learned a great deal.

This brief article is a report on the activities during that 25th course, as well as an opportunity to celebrate the importance of the Ceppellini School to connect young immunologists with leader scientists in their fields, as well as to spur new collaborations. With the generous support of the EFIS-EJI, the Bill and Melinda Gates Foundation, and the International Union of Immunological Societies, a record number of travel fellowships was offered to 13 participants from African countries, including South Africa, Kenya, Nigeria, Gabon, and Cameroon. This was appropriate because it is in Sub-Saharan Africa (SSA) that maternal morbidity and mortality is highest due to pregnancy complications, such as the hypertensive disorder of pregnancy pre-eclampsia, still birth or intrauterine growth restriction (3).

## Activities During the 2014 Course

On the first day of the course, Silvia Fontana Zappacosta talked about the ethos and history of the School founded by her late husband Serafino Zappacosta. One of the remits of the School is to “foster wider interest for immunology and to attract to the discipline young scientists, also from disadvantaged countries” (4). I introduced the course with a brief synopsis of each lecturer's topic and told the story of my own connection to the Ceppellini School. My late maternal uncle Tommaso (Tommi) Meo trained with Ceppellini himself in the 60's and 70's in Turin and Basel. Ceppellini made seminal contributions for the advancement of our understanding of immunogenetics (5). Among the factors determining pregnancy outcome are immune system genes—that is combinations of certain variants of genes coding for Human Leukocyte Antigens (HLA) and Killer-cell Immunoglobulin-like Receptors (KIR) (6). How odd that part of my research today was the subject of my uncle's science with Ceppellini and that he so excitedly narrated to us on his summer visits back in our native Southern Italy. John Trowsdale (University of Cambridge) reviewed the “ABC” of KIR and HLA, explaining how the system may have evolved to deploy the two A and B haplotypes that code for KIR receptors on Natural Killer (NK) cells to bind HLA-C on fetal trophoblast cells. Ashley Moffett (University of Cambridge) discussed how the KIR and HLA systems may have evolved and can be used to study population history (7). Annettee Nakimuli (Makerere University and Mulago Hospital, Kampala, Uganda) discussed the diverse KIR and HLA genes that cause susceptibility to or protection from pregnancy disorders in Europeans and Africans (8). Allison Elliott (London School of Hygiene and tropical Medicine and Uganda Virus Research Institute, Entebbe, Uganda) presented fascinating data on the impact of helminth infection during pregnancy and the outcomes in the offspring. Angela Santoni (University of Rome La Sapienza) reviewed leukocyte trafficking and the changes occurring during pregnancy. On the second day, Elizabeth Simpson (Imperial College, London, UK) gave a historical background on multiple histocompatibility antigens and how the maternal immune system is aware of fetal antigens yet does not mount an immune response against the fetus. Tamara Tilburgs (Harvard University, Cambridge, US and now at the Cincinnati Children's Hospital, US) discussed the delicate balance that the maternal immune system must strike between fetal tolerance and antiviral immunity. Jakob Michaelsson (Karolinksa Institut, Stockholm, Sweden) reminded the audience that the fetus also has its immune system that may engage with maternal antigens, with consequences on micro-chimerism. Marise Alegre (University of Chicago, US) revised the evidence that tolerance can be induced experimentally to transplants. Anthony De Tomaso (University of California Santa Barbara, US) talked about the strange and fascinating life of a basal chordate that uses allorecognition to regulate stem cell parasitism. In the third and last day, Ennio Carbone (Karolinksa Institute, Stockholm, Sweden and University of Catanzaro, Italy) opened the lectures with an overview on tumor immunology. Tom Gajewski (University of Chicago, US) followed up highlighting the immune pathways in the tumor microenvironment that may be operating also at the maternal-fetal interface, with the engagement of several inhibitory checkpoints. The course ended with my closing lecture on mouse models of immunogenetics of pregnancy.

In the typical spirit of the Ceppellini School, the presentations were enriched by ample discussions and debates in which both faculty and students participated actively. The search for elusive pathogenic T cells in pregnancy complications was discussed as it was the antigen specificity of these effector T cells, which most likely are HLA-C-restricted. Another theme was the importance of studying human populations in which the prevalence of pregnancy complications is highest. New technology that can help visualize lymphocytes at the maternal-fetal interface were discussed, including imaging approaches. Finally, various routes of vertical transmission were considered, including through maternal monocytes and fetal placental macrophages (i.e., Hofbauer cells).

## Fostering Young Immunologists and Facilitating Collaborations

There were plenty of opportunities for the participants to interact among each other and with the faculty members over lunches, coffee breaks, and the poster session. Several collaborations stemmed from this course and continue till today. Both Anthony De Tomaso and Allison Elliott, two of the members of the faculty at this course came to spend a year as visiting Fellows of King's College, Cambridge, where myself and Ashley Moffett are also Fellows. Annette Nakimuli and Ashley Moffett have strengthened their collaboration and have since initiated a series of initiatives within the Cambridge-Africa partnership to improve patients care in the Department of Obstetrics and Gynecology at the Makerere University, including several trips from Cambridge obstetricians to visit Uganda. For example, Catherine Aiken, also at our Department, now mentors Imelda Namagembe's PhD thesis in Uganda, that focuses on improving maternal health.

Despite not present at the course, Stephen Tukwasibwe, then a research assistant in the same hospital of faculty member Annettee Nakimuli, became interested in the immunogenetics of pregnancy. Having worked successfully on the genetics of resistance to malaria and secured a Wellcome Trust PhD grant, Stephen started his thesis at Makerere University under the supervision of Annettee Nakimuli and my co-mentorship, to test the hypothesis that *Plasmodium* may have selected for those genetic variants that may protect from malaria but expose women to pregnancy complications in SSA. Stephen has since visited Cambridge several times working at the Pathology Department as part of his thesis. One of the participants, Iva Filipovic from Serbia, was completing her MSc degree at Imperial College, London, during the course and was very keen to learn more on immunology of pregnancy. She secured a PhD Studentship from the University of Cambridge Center for Trophoblast Research and came to work on her PhD as a graduate student of King's College and in my laboratory to study the gene expression profile of innate lymphoid cells in the uterus of mice (9). She is currently working as a post-doc at the Karolinska Institute and I look forward to seeing her future successes.

## New Concepts and Recent Progress in the Field

Peter Medawar in 1953 famously proposed three mechanisms underlying placental tolerance: (i) anatomical separation of mother and fetus; (ii) antigenic immaturity of the fetus; (iii) immunological unresponsiveness of the mother. Bearing in mind these proposals were formulated in light of the progress made during those days in transplantation immunology, and with unimaginable less knowledge of the details of the human immune system then we have today, it is perhaps not surprising that none of these three mechanisms have been fully substantiated—although they have influenced generations of immunologists of reproduction. On the contrary, we know that the placenta is not such a tight barrier and cells can mix in both directions. We also know that the fetus is not antigenically immature and the mother is not unresponsive. Indeed, pregnant women can make both T cells and antibodies that recognize fetal antigens (e.g., anti-D antibodies in Rhesus incompatibility).

One major conceptual shift in the immunology of pregnancy is the understanding that pregnant women are not immunosuppressed. Changes in the immune system during pregnancy may however be responsible for the greater morbidity and mortality of mothers and infants infected with certain pathogens (10). The emergence of new epidemics has attracted the attention of investigators who are now addressing the mechanisms of vertical transmission of certain pathogens, e.g., Zika virus (11, 12). That microbes are integral part of human health and disease has become established in the recent past, perhaps best illustrated by the influence of the gut microbiota on the immunotherapy of cancer (13)—one of themes of the 2019 course (*Microbes, Immunity and Cancer*) of the Ceppellini School (14). Transplantation immunology also may be influenced by microbes (15, 16), however the search for a placental microbiome has so far been elusive (17). Yet, maternal infections may have repercussions on neuropsychiatric disorders (18) and the development of the immune system in the offspring. Clinical trials are ongoing to evaluate the effectiveness of vaccinating mothers to prevent children's allergies (19, 20).

There are obvious selective disadvantages in a strategy that would suppress the immune system of pregnant women to allow the implantation and growth of the placenta. The placenta evolved much later than the immune system and it is reasonable to think that placentation and immunity have co-evolved agreeably, rather than embarking in a deleterious conflict. One illustrative example may be the interactions of maternal KIR on uterine NK cells with fetal HLA-C molecules on the placental cells, which may engage in a molecular conversation that, rather than leading to allorecognition-driven rejection, may in fact contribute to uterine vascular remodeling and placental growth (6). Adding to the complexity of the maternal-fetal interactions is the heterogeneity of immune cells, revealed recently by singe-cell RNA-sequencing (21) and mass cytometry (22). Mass cytometry has been applied to study also the fluctuations in blood immune cells throughout pregnancy (23, 24). Multiple populations of innate lymphoid cells (9, 21, 25), regulatory T cells (26), and macrophages (27) compose the diverse immune cell landscape operating at the maternal-fetal interface, which varies during the stages of pregnancy and it is therefore difficult to decipher precisely. New technology such as three-dimensional organoid cell cultures (28) may help to determine some of the mechanisms underlying placentation (29). Advances in typing polymorphic KIR and HLA genes (30, 31) may also help to shed light on the immunogenetics of pregnancy. Although the interactions of maternal KIR with fetal HLA-C may be a pivotal one to activate uterine NK cells and determine the outcome of pregnancy (6), the importance of the interaction of NK cell receptors with self HLA class I molecules is emerging, in a process known as NK-cell education. We have shown recently that NK-cell education in the uterus may follow different rules than in the blood (32) and that NK-cell education reduces the risk of pregnancy complications in women genetically programmed to engage the inhibitory NKG2A receptor on NK cells (33). The next grand challenge is to precisely decipher the multiple and changing interactions between mother and fetus in the decidua, to eventually manipulate them in order to improve the outcome of pregnancy (29).

## Author Contributions

The author confirms being the sole contributor of this work and has approved it for publication.

### Conflict of Interest

The author declares that the research was conducted in the absence of any commercial or financial relationships that could be construed as a potential conflict of interest.
